# Attenuation of sleep deprivation dependent deterioration in male fertility parameters by vitamin C

**DOI:** 10.1186/s12958-020-0563-y

**Published:** 2020-01-11

**Authors:** Nermin I. Rizk, Mohamed S. Rizk, Asmaa S. Mohamed, Yahya M. Naguib

**Affiliations:** 1 Clinical Physiology Department, Faculty of Medicine, Menoufia University, Menoufia, Egypt; 2 Medical Biochemistry and Molecular Biology Department, Faculty of Medicine, Menoufia University, Menoufia, Egypt; 3 Pathology Department, Faculty of Medicine, Menoufia University, Menoufia, Egypt

**Keywords:** Male fertility, Sleep deprivation, Stress, Spermatogenesis, Oxidative stress, Nrf2

## Abstract

**Purpose:**

Male fertility is multifaceted and its integrity is as well multifactorial. Normal spermatogenesis is dependent on competent testicular function; namely normal anatomy, histology, physiology and hormonal regulation. Lifestyle stressors, including sleep interruption and even deprivation, have been shown to seriously impact male fertility. We studied here both the effects and the possible underlying mechanisms of vitamin C on male fertility in sleep deprived rats.

**Methods:**

Thirty male Wistar albino rats were used in the present study. Rats were divided (10/group) into: control (remained in their cages with free access to food and water), sleep deprivation (SD) group (subjected to paradoxical sleep deprivation for 5 consequent days, rats received intra-peritoneal injections of vehicle daily throughout the sleep deprivation), and sleep deprivation vitamin C-treated (SDC) group (subjected to sleep deprivation for 5 consequent days with concomitant intra-peritoneal injections of 100 mg/kg/day vitamin C). Sperm analysis, hormonal assay, and measurement of serum oxidative stress and inflammatory markers were performed. Testicular gene expression of Nrf2 and NF-κβ was assessed. Structural changes were evaluated by testicular histopathology, while PCNA immunostaining was conducted to assess spermatogenesis.

**Results:**

Sleep deprivation had significantly altered sperm motility, viability, morphology and count. Serum levels of cortisol, corticosterone, IL-6, IL-17, MDA were increased, while testosterone and TAC levels were decreased. Testicular gene expression of Nrf2 was decreased, while NF-κβ was increased. Sleep deprivation caused structural changes in the testes, and PCNA immunostaining showed defective spermatogenesis. Administration of vitamin C significantly countered sleep deprivation induced deterioration in male fertility parameters.

**Conclusion:**

Treatment with vitamin C enhanced booth testicular structure and function in sleep deprived rats. Vitamin C could be a potential fertility enhancer against lifestyle stressors.

## Introduction

Infertility is a reasonably common condition with medical, psychological, and financial consequences. Infertility can be defined as the inability of a couple to conceive after 1 year of attempting conception. Infertility affects an estimated 15% of couples worldwide, of which, male are considered to be solely responsible for 20–30% of infertility cases [1]. Accumulating data suggest that there is a progressive reduction in human sperm quality and 50–60% reduction in sperm counts in men in recent decades [2]. Male infertility can be influenced by environmental, occupational, and modifiable lifestyle factors such as psychological stress, obesity, smoking, mobile phone radiations and lack of sleep [3, 4].

Sleep is a physiological periodic state of rest. Sleep is a bio-vital phenomenon that is associated with neuro-endocrin and immunity changes [5]. Adequate sleep is a basic inquiry for healthy life and proper fertility; there is a strong correlation between adequate sleep and gonadotropin releasing hormone (GnRH) secretion which plays a basic role in the productive functions [6]. Furthermore, adequate sleep positively affects the sexual behaviour. It was reported that increasing the night sleep 1 h enhances the sexual activity by 14% [7]. Sleep deprivation (SD) is a common social stress that affects a wide range of population. According to the National Sleep Foundation, there is a marked increase in the incidence of sleep deprivation in the last few years. Night shift workers and patient suffering psycho-social disturbances are the most vulnerable populations. SD involves a wide range of disorders such as; behaviourally induced insufficient sleep syndrome, sleep apnea and insomnia [8].

SD has serious adverse effects on different body functions resulting in cardiovascular diseases, immune disturbances and neuro-endocrinal changes [9]. Furthermore, SD and psychological stress alter the activity of the hypothalamic-pituitary-adrenal (HPA) axis and the sympathetic nervous system with negative impact on both sexuality and fertility [10]. Inadequate sleep has been reported to decrease semen quality [11]. Previously published studies have shown that the immune function could be impaired by sleep deprivation [12]. The level of the immunoglobulins G, A and M was enhanced in a sleep deprived cohort study, suggesting that the serum humoral immunity parameters could be altered following insufficient sleep [13]. It was reported that short sleep duration, long sleep duration and late bedtime impair semen quality partly via the increased production of seminal anti-sperm antibody [14]. One could produce antibodies to his own sperms in certain conditions such as varicocele [15], intercourse [16], as well as testicular inflammation [17]. Spermatogenesis is an active replecative process generating about 1000 sperm/second. The high rate of cell division requires rationally higher mitochondrial oxygen consumption [18]. Under stressful conditions, spermatozoa generate small amounts of reactive oxygen species (ROS). Minimal amounts of ROS are essential for acrosomal reaction and fertilization, however, excessive production of ROS can cause damage of normal spermatozoa through lipid peroxidation and DNA damage [19]. Testicular membrane is rich in polyunsaturated fatty acids (PUFA) rendering the testes vulnerable to lipid peroxidation and eventually oxidative stress injury [18].

Great attention has been given to molecules with potentially polymodal protective effects. Vitamin C, ascorbic acid, is present in the testes presumably playing a pivotal role in the testicular antioxidant defence system and, therefore, supporting spermatogenesis. However, in order to function effectively as an antioxidant, vitamin C must be maintained at high levels in the body [13]. In addition, vitamin C has potential anti-inflammatory properties; vitamin C has been reported to alleviate the inflammatory status by reducing hsCRP and IL-6 in hypertensive and/or diabetic obese patients [20]. On the basis of these considerations, the aim of the present study was to test the hypothesis that vitamin C could counteract the detrimental effects of SD on male fertility. To achieve that, we examined the effect of vitamin C administration on semen quality, reproductive hormones, oxidative and inflammatory markers, testicular structure, and testicular expression of genes contributing to oxidative and inflammatory homeostasis in sleep-deprived adult male rats.

## Materials and methods

### Animals

Thirty male Wistar albino rats were used in the present study. The experimental procedures were conducted in adherence to the Guiding Principles in the Use and Care of Animals published by the National Institutes of Health (NIH Publication No 85–23, Revised 1996). Animal care and use was approved by the Menoufia University Ethics Committee. Animals were kept for 10 days prior to the start of the study to allow proper acclimatization. The animals were fed standard laboratory chow and allowed free access to water in an air-conditioned room with a 12 h light-dark cycle.

### Animal groups

Following acclimatization, rats were assigned randomly into three experimental groups of 10 rats each:

1. Control group (C): rats remained in their cages with free access to water and balanced diet.
2. Sleep deprivation group (SD): rats were subjected to paradoxical sleep deprivation for 5 consequent days. Rats had water and food ad libitum during the sleep deprivation period. Rats received intra-peritoneal injections of vehicle daily throughout the sleep deprivation phase.
3. Sleep deprivation + vitamin C-treated group (SDC): rats were subjected to sleep deprivation for 5 consequent days with concomitant intra-peritoneal injections of 100 mg/kg/day vitamin C (20% vials, Global Cosmetic Solutions, SL, Spain). Rats had water and food ad libitum during the sleep deprivation period.

### Sleep deprivation

Sleep deprivation was induced according to the method of Choi et al., 2016 with slight modifications [9]. Rats were kept in a custom-made glass tank (120 × 40 × 40 cm) containing 10 platforms. The platforms were carefully designed to allow alert standing of each rat, but do not allow them to sleep. When rats tend to fall asleep, they lose their balance; hence they fall in water and awaken. Animals could move only by jumping from one platform to another. Before filling the glass tank with water, rats were left in the glass tank 1 h/day for 3 consequent days for acclimatization. After the acclimatization period, the glass tank was filled with water 3 cm beneath the surface of the platforms.

### Blood sample collection

At the end of the study, all rats were fasted overnight. Blood was drawn from each rat via cardiac puncture. The blood was allowed to coagulate for 30 min at room temperature. Blood samples were then centrifuged at 4000 rpm for 15 min to separate serum samples. Serum samples were stored at − 20 °C. Finally, all rats were scarified by cervical dislocation.

### Biochemical assessment

Serum levels of cortisol (BioVision, USA), testosterone (CUSABIO, Shanghai, Chaina), interleukin 17 (IL-17, Abcam, USA), and interleukin 6 (IL-6, Abcam, USA) were determined by quantitative sandwich enzyme immunoassay technique using an automatic optical reader (SUNRISE Touchscreen, TECHAN, Salzburg, Austria). Malondialdehyde (MDA) and total antioxidant capacity (TAC) (Abcam, USA) were determined by routine kinetic and fixed rate colorimetric methods on a Jenway Genova autoanalyser (UK).

### Evaluation of testicular GSH, MDA and GPx

Rats were sacrificed by cervical dislocation. Both testes were dissected, weighed and then washed with cold saline. The left testes were homogenized in lysis buffer solution (abcam, USA, 1:5 w/v). The homogenate was centrifuged and the supernatant was used for colorimetric estimation of glutathione (GSH, QuantiChrom™, BioAssay Systems, USA), glutathione peroxidase (GSH-Px, EnzyChrom™, BioAssay Systems, USA) and MDA tissue levels using fixed rate colorimetric method.

### Collection of semen

Cauda epididymis was dissected free in a Petri dish containing 5 ml warm saline solution (37 °C). Then, it was cut into pieces by a fine medical scissor and incubated for 5 min with frequent shaking to yield semen suspension. Semen suspension was used for further assessment of sperm motility (%), viability (%), abnormal forms (%) and total sperm count (in millions) [9].

### Assessment of sperm motility

After 5–10 min of dissection, a drop of semen suspension was smeared on a glass slide and examined under light microscope (power 400X) to assess sperm motility. A minimum of three different fields were examined to determine the mean percentage of sperm motility [21].

### Sperm viability

Equal volumes (100 μl) of semen suspension and eosin stain (1%) were mixed and incubated for 2 min. A drop of this mixture was smeared on a clean glass slide and examined under light microscope. Living sperms were not affected by the stain, while dead sperms stained pink by eosin [22]. The percentage of viability was determined in the field examined (number of alive sperm/total number sperm*100). In each sample, a minimum of three different fields were examined to determine the mean percentage of sperm viability.

### Assessment of sperm morphology

A drop of semen suspension was smeared on a glass slide and examined by light microscope (power 400X). The percentage of abnormal forms, in each field, was determined (number of abnormal sperms/total number of sperms*100). Ten fields were examined in each slide to determine the mean percentage of abnormal forms [23].

### Sperm count

Ten microliter of the semen suspension was smeared on the counting haemocytometer. Sperm counting was done under light microscope (200X). The results were expressed as million/ml of suspension. The sperm count was repeated at least twice and the average was taken. Total sperm count was calculated as (Count*dilution* 5*10^4^) [24].

### Histopathology examination

Specimens from the right testes were fixed in 10% formol saline for 5–7 days. The specimens were washed in tap water for 10 min and then dehydrated in graded ethanol solutions (70, 90% over night and 100% ethanol solution for three changes 1 h each). The specimens were cleared in xylene (20–30 times). After that, specimens were impregnated in soft paraffin wax at 55–60 °C for 2 h then in hard paraffin wax at room temperature in moulds. Tissue blocks were cut into section of 5 μm thickness by using rotator microtome. Tissue sections were dipped in a warm water-bath, picked up on clean slides, and placed on hot plate for 2 min. Finally, tissue sections were stained with haematoxylin and eosin stain for general architecture of the studied tissues.

### PCNA immunostaining

Immunostaining staining was carried out using primary antiserum to proliferating cell nuclear antigen (PCNA) (PC10, Santa Cruz Biotechnology Inc., Heidelberg, Germany). Briefly, the primary antibody was diluted in Tris buffer with a dilution of 1:50 (as determined by the data sheet). The sections were incubated with the primary antibody overnight at + 4 °C. The binding of the primary antibody was observed using a commercial avidinbiotin- peroxidase detection system recommended by the manufacturer (DAKO, Carpenteria, USA). Finally, the slides were stained with diaminobenzene (DAB).

### Analysis of gene expression by quantitative RT-PCR (qRT-PCR)

Real time quantitative reverse transcription-polymerase chain reaction (RT-PCR) assay was used to examine mRNA expression of nuclear factor (erythroid-derived 2)-like 2 (Nrf2) and nuclear factor kappa beta (NF-κβ) genes in the studied groups. To extract RNA, frozen testicular specimens were ground using a mortar and pestle and liquid nitrogen. Total RNA was extracted with TRI reagent (Sigma-Aldrich, New South Wales, Australia). To generate the template for PCR amplification, 2 μg of testicular RNA was reverse transcribed into cDNA using the high capacity RNA-to-cDNA kit (Applied Biosystems, Foster City, CA, USA). This cDNA was used to determine the mRNA expression for the genes of interest by quantitative real-time PCR as previously described using gene specific primers (Table 1), designed using Primer Express Software version 2.0 (Applied Biosystems, Victoria, Australia). GAPDH was used as the housekeeping control loading gene. SYBR green PCR assays for each target molecule and internal reference GAPDH were performed in duplicate on these cDNA samples in a 10 μL reaction using Applied Biosystems 7500 FAST 96-well PCR machine. From the amplification curves, relative expression was calculated using the comparative Ct (2 − ΔCt) method, with glyceraldehyde-3-phosphate dehydrogenase (GAPDH) serving as the endogenous control and the expression data as a ratio (target gene/GAPDH).

**Table 1:** Sequence of the primers used for assessing Nrf2 and NF-κβ gene expression

Nrf2	Forward	CACATCCAGACAGACACCAGT
	Reverse	CTACAAATGGGAATGTCTCTGC
NF-κβ	Forward	CGCCACCGGATTGAAGAAAA
	Reverse	TTGATGGTGCTGAGGGATGT
GAPDH	Forward	TGCACCACCAACTGCTTAGC
	Reverse	GGCATGGACTGTGGTCATGAG

### Statistical analysis

Kolmogorov-Smirnov test was performed on all data sets to ensure normal distribution (*p* > 0.5). Results are expressed as mean ± standard deviation (SD). Analyses of Variances (ANOVA) with Tukey’s honesty significant difference (HSD) tests were used for statistical analysis using Origin® software and the probability of chance (*p* values). *P* values < 0.05 were considered significant.

## Results

**Fig. 1:**
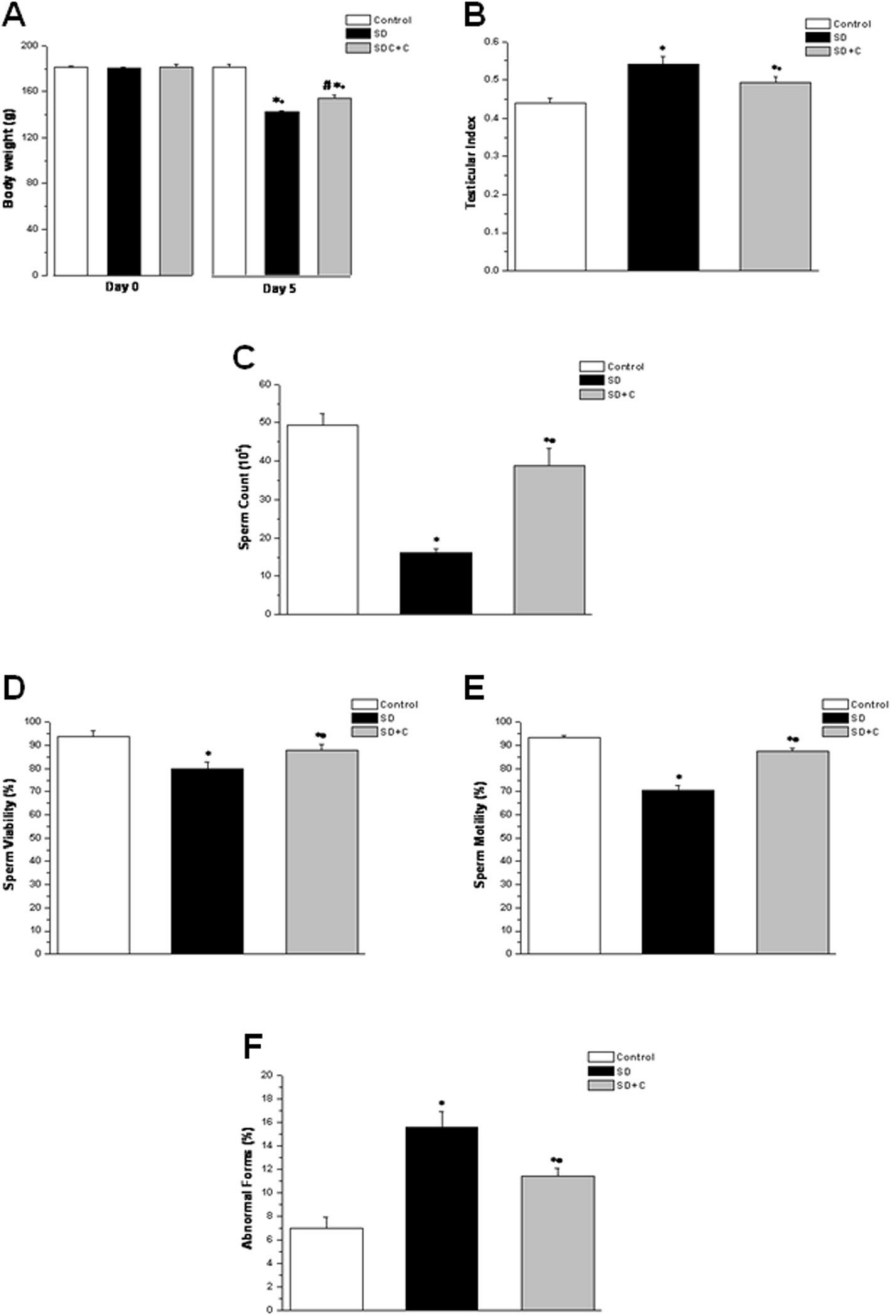
Effect of vitamin C on body weight, testicular index and sperm evaluation in sleep deprived rats. **a** Body weight in control (white column), sleep deprived (black column) and sleep deprived + vitamin C treated (grey column) groups. **b** Testicular index in control (white column), sleep deprived (black column) and sleep deprived + vitamin C treated (grey column) groups. **c** Sperm count in control (white column), sleep deprived (black column) and sleep deprived + vitamin C treated (grey column) groups. **d** Sperm viability in control (white column), sleep deprived (black column) and sleep deprived + vitamin C treated (grey column) groups. **e** Sperm motility in control (white column), sleep deprived (black column) and sleep deprived + vitamin C treated (grey column) groups. **f** Abnormal forms in control (white column), sleep deprived (black column) and sleep deprived + vitamin C treated (grey column) groups. (Significant = *p* < 0.05, * significant when compared to the control group, • significant when compared to the sleep deprived group, # significant when compared to the same group at the start of the experiment. Number of rats = 10/group)

Although there was no significant difference in body weight between the experimental groups at the start of the experiment; the body weight was significantly lower in the SD group when compared to the control group (142.31 ± 0.89 g vs. 181.66 ± 1.85 g, *p* < 0.05) following 5 days of sleep deprivation. Interestingly, administration of vitamin C resulted in significant increase in body weight in the in SDC group (153.99 ± 3.02 g, *p* < 0.05) when compared to the SD group, albeit it was still significantly lower if compared to the corresponding values in the control group, or the SDC group itself at the start of the experiment (Fig. 1a). Unsurprisingly, there was significant increase in the testicular index in the SD group when compared to the control group (0.54 ± 0.018 vs. 0.44 ± 0.012, *p* < 0.05), while there was significant reduction in the SDC group (0.492 ± 0.016, *p* < 0.05) when compared to SD group. Testicular index was significantly higher in the SDC group when compared to the control group (Fig. 1b).

As shown in Fig. 1c, d, e, and f, there was significant decrease in sperm count, viability and motility, with significant increase in abnormal forms of sperms in SD group when compared to the control group (16.15 ± 1.07 × 10^6^, 70.6 ± 1.96%, 80 ± 2.74% and 15.6 ± 1.33% vs. 49.42 ± 2.88 × 10^6^, 93.2 ± 0.97%, 94 ± 2.45% and 7 ± 0.95% respectively, *p* < 0.05). Sperm count, viability and motility were significantly higher, while the abnormal forms of sperms were significantly lower in the SDC group (38.86 ± 4.34 × 10^6^, 87.6 ± 1.12%, 88 ± 2.55% and 11.4 ± 0.68% respectively, *p* < 0.05) compared to the SD group. However, sperm count, viability and motility still significantly lower and abnormal forms still significantly higher in SDC group when compared to control group.

**Fig. 2:**
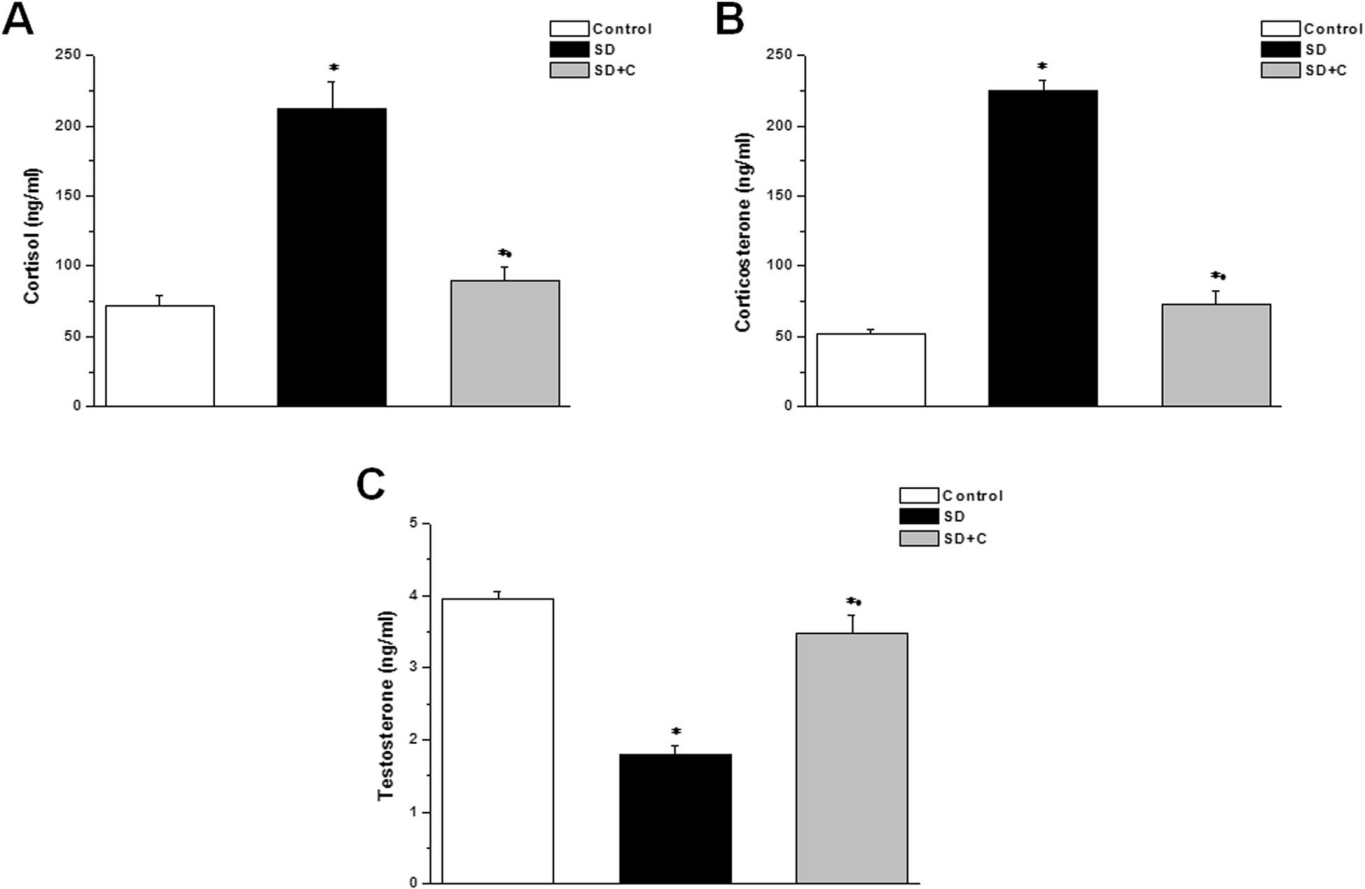
Effect of vitamin C on serum hormones in sleep deprived rats. **a** Cortisol level in control (white column), sleep deprived (black column) and sleep deprived + vitamin C treated (grey column) groups. **b** Corticosterone level in control (white column), sleep deprived (black column) and sleep deprived + vitamin C treated (grey column) groups. **c** Testosterone level in control (white column), sleep deprived (black column) and sleep deprived + vitamin C treated (grey column) groups. (Significant = *p* < 0.05, * significant when compared to the control group, • significant when compared to the sleep deprived group. Number of rats = 10/group)

Serum cortisol and corticosterone levels were significantly higher, while testosterone level was significantly lower in the SD group when compared to the control group (212.58 ± 18.44 ng/ml, 224.6 ± 8.12 ng/ml and 1.79 ± 0.14 ng/ml vs. 72.15 ± 6.98 ng/ml, 52.4 ± 3.17 ng/ml and 3.95 ± 0.11 ng/ml respectively, *p* < 0.05). In the vitamin C treated sleep-deprived rats, serum cortisol and corticosterone levels were significantly lower, while testosterone level was higher (90.43 ± 9.35 ng/ml, 73.48 ± 9.36 ng/ml and 3.48 ± 0.25 ng/ml respectively, *p* < 0.05) when compared to the SD group. However, cortisol and corticosterone levels were significantly higher and testosterone level was significantly lower in the SDC group when compared to the control group (Fig. 2).

**Fig. 3:**
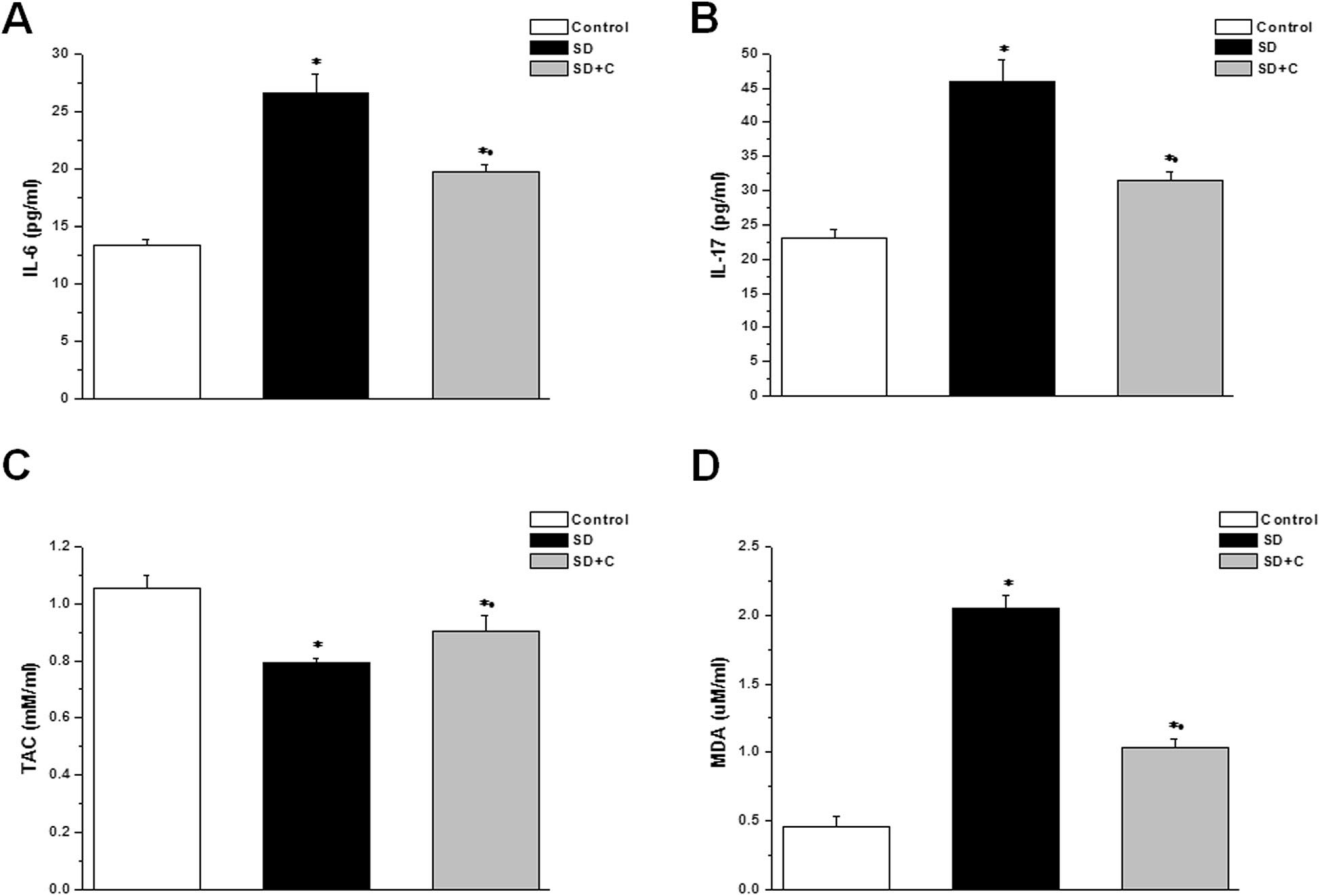
Effect of vitamin C on serum inflammatory and oxidative stress markers in sleep deprived rats. **a** IL-6 level in control (white column), sleep deprived (black column) and sleep deprived + vitamin C treated (grey column) groups. **b** IL-17 level in control (white column), sleep deprived (black column) and sleep deprived + vitamin C treated (grey column) groups. **c** TAC in control (white column), sleep deprived (black column) and sleep deprived + vitamin C treated (grey column) groups. **d** MDA level in control (white column), sleep deprived (black column) and sleep deprived + vitamin C treated (grey column) groups. (Significant = *p* < 0.05, * significant when compared to the control group, • significant when compared to the sleep deprived group. Number of rats = 10/group)

Serum levels of IL-6 and IL-17 were significantly higher in the SD group when compared to the control group (26.6 ± 1.6 pg/ml and 46.1 ± 3.16 pg/ml vs. 13.4 ± 0.51 pg/ml and 23 ± 1.41 pg/ml respectively, *p* < 0.05). IL-6 and IL-17 levels were significantly lower in the SDC group (19.8 ± 0.58 and 31.4 ± 1.33 pg/ml) when compared SD groups, yet they were still significantly higher than the corresponding values in the control group (Fig. 3a and b). Sleep deprivation resulted in significant reduction in the TAC and significant elevation in MDA levels when compared to the control group (0.79 ± 0.01 mM/ml and 2.05 ± 0.09 uM/ml vs. 1.05 ± 0.05 mM/ml and 0.46 ± 0.07 uM/ml respectively, *p* < 0.05). TAC was significantly higher and MDA was significantly lower in the SDC group (0. 9 ± 0.05 mM/ml and 1.04 ± 0.06 uM/ml respectively, *p* < 0.05) when compared to the SD group, while the TAC remained significantly lower with significantly higher MDA levels in the SDC group if compared to the control group (Fig. 3c and d).

**Fig. 4:**
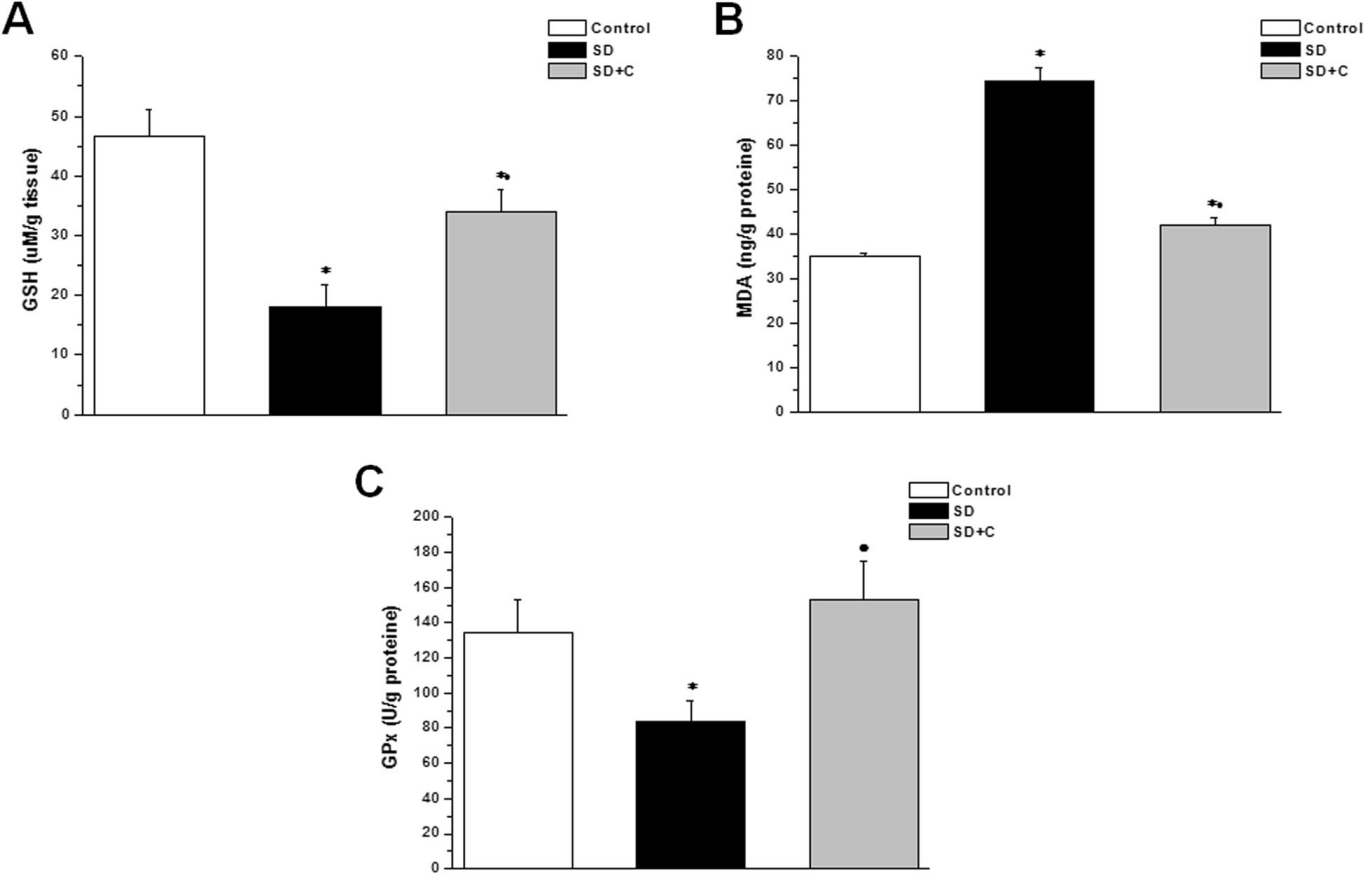
Effect of vitamin C on testicular oxidative-antioxidative parameters in sleep deprived rats. **a** GSH level in control (white column), sleep deprived (black column) and sleep deprived + vitamin C treated (grey column) groups. **b** MDA level in control (white column), sleep deprived (black column) and sleep deprived + vitamin C treated (grey column) groups. **c** GPx level in control (white column), sleep deprived (black column) and sleep deprived + vitamin C treated (grey column) groups. (Significant = *p* < 0.05, * significant when compared to the control group, • significant when compared to the sleep deprived group. Number of rats = 10/group)

Testicular GSH and GPx tissue levels were significantly lower, while testicular MDA was significantly higher in the SD group when compared to the control group (18.15 ± 3.63 uM/g tissue, 83.47 ± 12.33 U/g tissue and 74.37 ± 2.93 ng/g tissue vs. 46.67 ± 4.41 uM/g tissue, 134.12 ± 18.79 U/g tissue and 34.81 ± 0.94 ng/g tissue respectively, *p* < 0.05). In vitamin C treated SD group, testicular tissue levels of GSH and GPx were significantly higher, while MDA tissue level was significantly lower (33.87 ± 3.92 uM/g tissue, 152.92 ± 21.72 U/g tissue and 42.02 ± 1.77 ng/g tissue respectively, *p* < 0.05) when compared to the SD groups. Testicular level of GSH was significantly lower, while MDA level was significantly higher in the SDC group, when compared to the control group (Fig. 4).

**Fig. 5:**
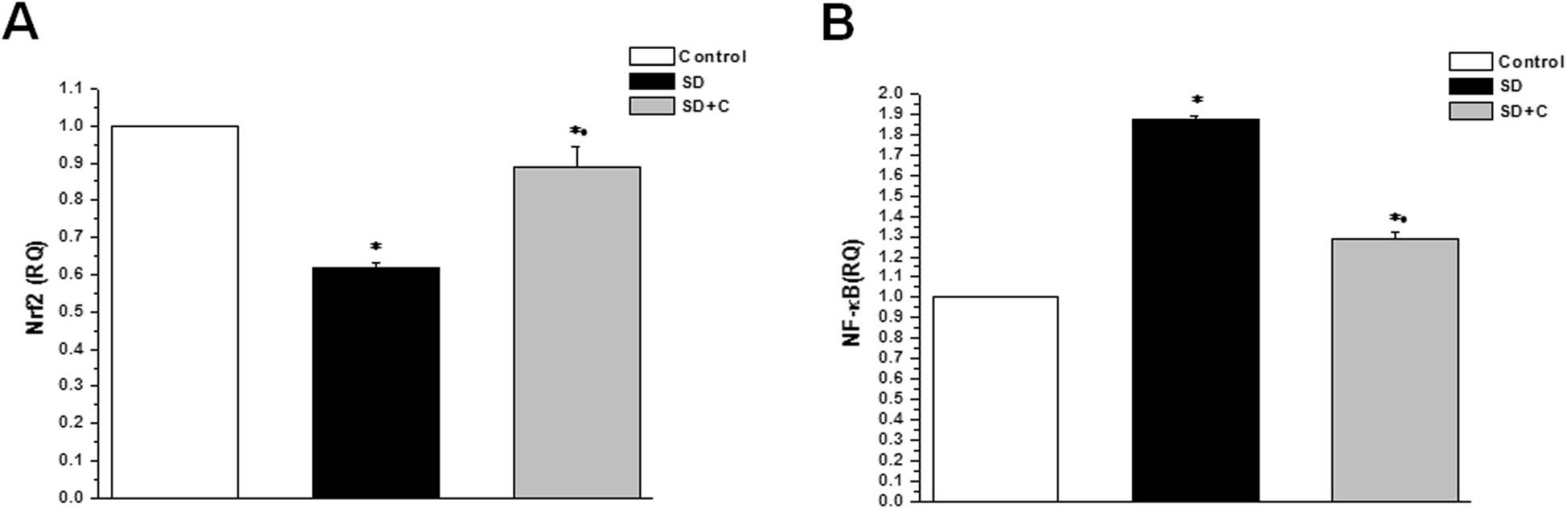
Effect of vitamin C on testicular Nrf2 and NF-κβ gene expression in sleep deprived rats. **a** Nrf2 gene expression in control (white column), sleep deprived (black column) and sleep deprived + vitamin C treated (grey column) groups. **b** NF-κβ gene expression in control (white column), sleep deprived (black column) and sleep deprived + vitamin C treated (grey column) groups. (Significant = *p* < 0.05, * significant when compared to the control group, • significant when compared to the sleep deprived group. Number of rats = 10/group)

Gene expression of Nrf2 in the testicular tissue was significantly downregulated, while gene expression of NF-κβ gene was significantly upregulated in the SD group when compared to the control group (0.62 ± 0.014 and 1.87 ± 0.02, vs. 1 RQ respectively, *p* < 0.05). Nrf2 gene expression was significantly higher, while NF-κβ gene expression was significantly lower in the SDC group (0.89 ± 0.06 and 1.29 ± 0.04 RQ respectively, *p* < 0.05) when compared to the SD group, however, the gene expression of Nrf2 remained significantly lower and NF-κβ significantly higher in the SDC if compared to the corresponding values in the control group (Fig. 5).

**Fig. 6:**
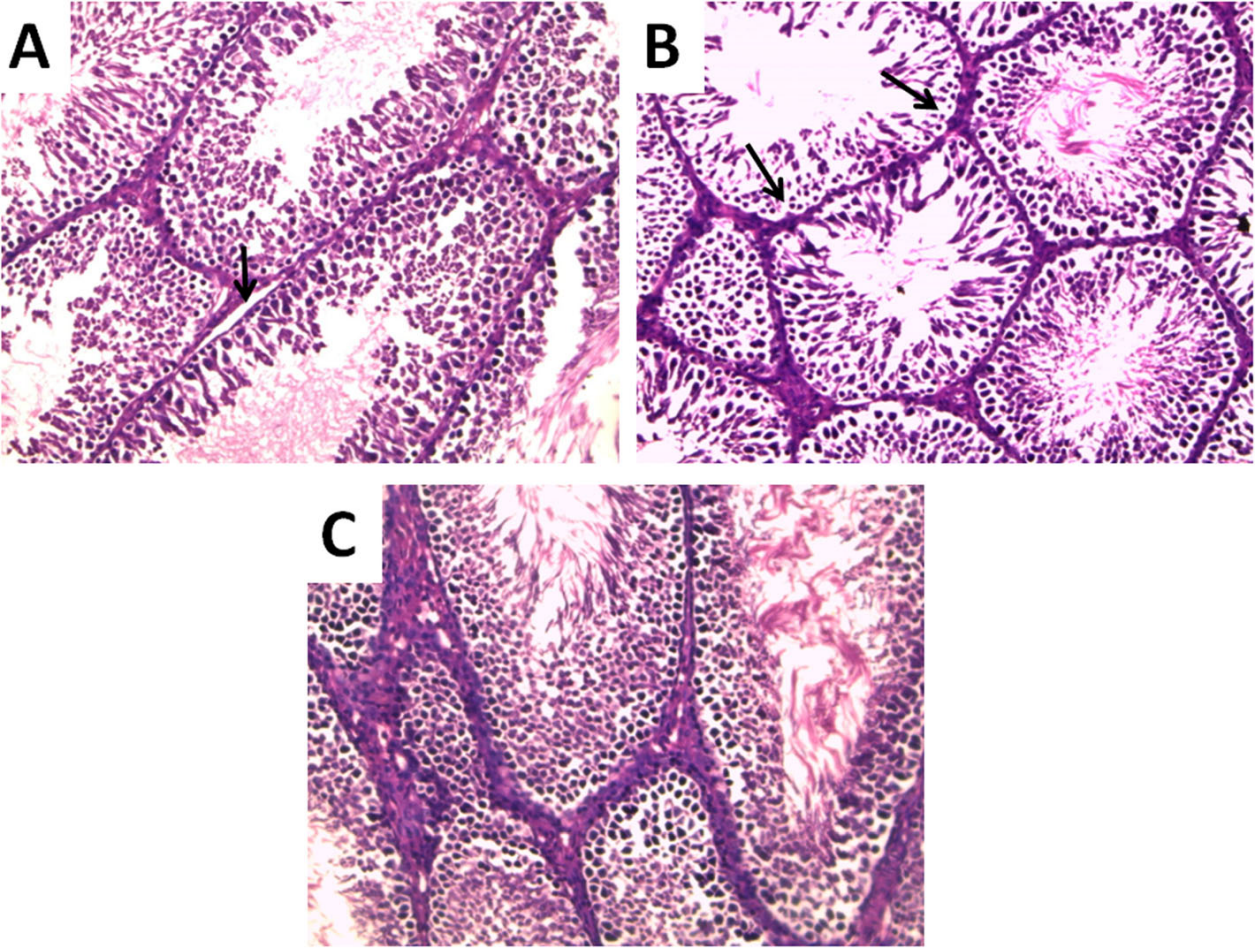
Vitamin C preserves testicular structure in sleep deprived rats. **a** Representative photomicrograph of Hx & E stained testis sections from control group showing normal oriented regular shaped seminiferous tubules with different stages of spermatogenesis, thin basement membrane (arrow) and interstitial spaces showing thin walled blood vessels (X 200). **b** Representative photomicrograph of Hx & E stained testis sections from sleep deprived group showing multiple seminiferous tubules with abnormal morphology of spermatogonia (arrows), shrunken nucleus and vacuolated cytoplasm), occasional apoptotic cells and very few late spermatids. Thick walled blood vessels can be seen in interstitial spaces (X 200). **c** Representative photomicrograph of Hx & E stained testis sections from sleep deprived + vitamin C treated group showing seminiferous tubules lined by multiple layers of spermatogonia and spermatocytes, with early and late spermatids filling the lumen(X 200). (Number of rats = 10/group)

**Fig. 7:**
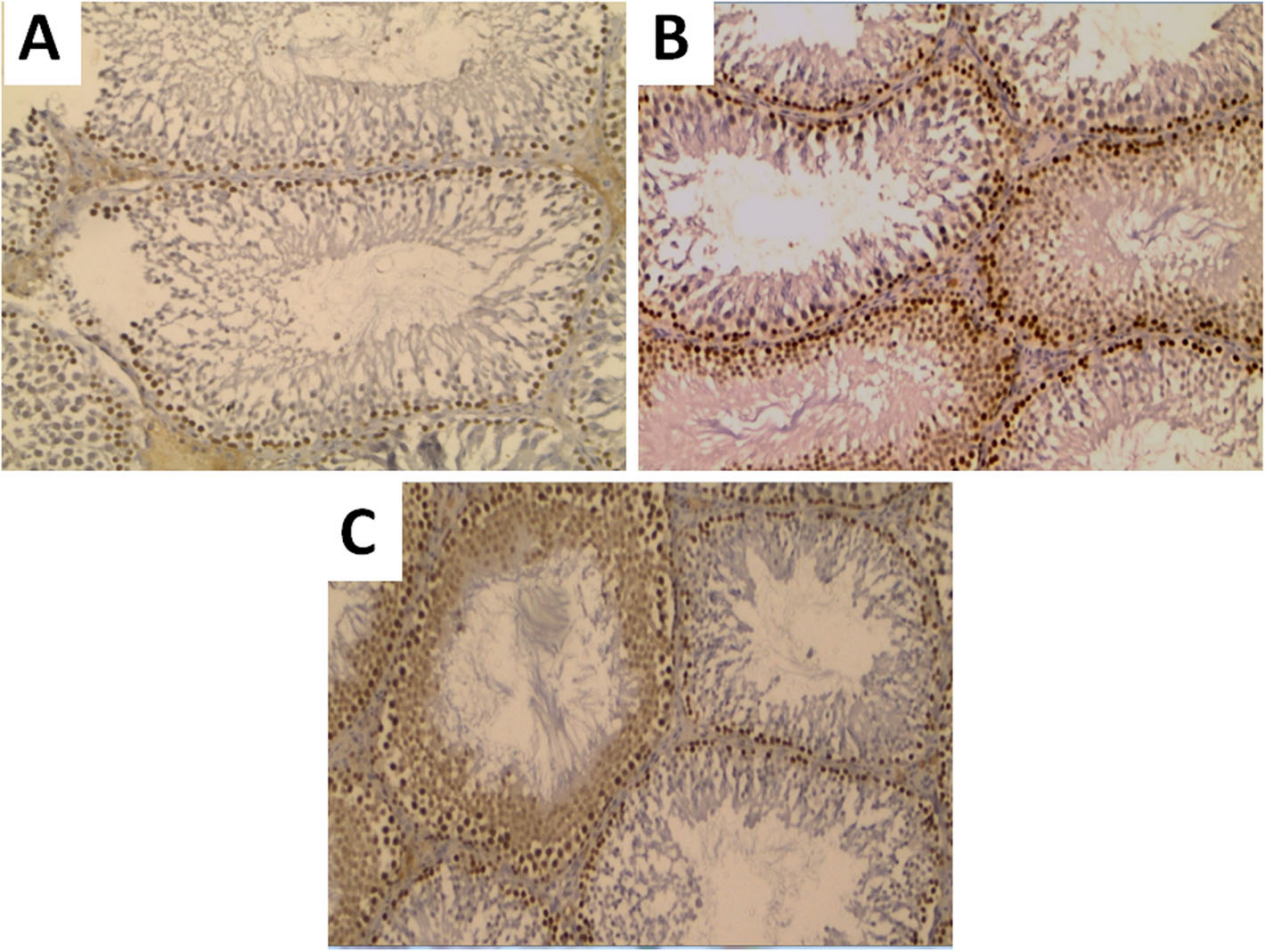
PCNA immunostaining in the studied groups. **a** Representative photomicrograph of PCNA immunostained sections in testis of control group showing strong positive staining of most of proliferating basal cells in seminiferous tubules. **b** Representative photomicrograph of PCNA immunostained sections in testis of sleep deprived group showing patchy positive staining of proliferating basal cells in seminiferous tubules **c** Representative photomicrograph of PCNA immunostained sections in testis sleep deprived + vitamin C treated group showing increased positive staining of proliferating basal cells in seminiferous tubules. (X 200, Number of rats = 10/group)

Histopathological evaluation of testicular biopsies revealed abnormal morphology of seminiferous tubules in the SD group with cellular degeneration of spermatogonia and thickening of the basement membrane. Interestingly, in the SDC group, more preserved architecture and morphology of spermatogonia were observed (Fig. 6). PCNA immunostaining revealed decreased positive immunostaining of the basal cell layer in the SD group when compared to the control group. Treatment with vitamin C resulted in enhancement of PCNA immunostaining when compared to the SD group, reflecting a qualitative improvement of spermatogenesis (Fig. 7).

## Discussion

Infertility is defined as inability of couples to conceive after 1 year of unprotected intercourse. Thereby, infertility affects 13–18% of couples, and male factor accounts for up to half of all the cases. Development of male infertility is influenced by many diseases and/or risk factors. Importantly, increase in risk of infertility can be noted, mostly in male population, when exposed to environmental stressors including sleep deprivation [25]. Better understanding of fertility and semen quality at the molecular levels in the male reproductive system could lead to a great achievement in treating infertility. Essentially, a better treatment for fertility and sexual dysfunction could improve the overall quality of life. Molecules with polymodal actions have gained much attention to minimize male reproductive tissue injuries, and enhance male fertility.

In the present study, sleep deprivation resulted in significant decrease in sperm count, viability and motility, while there was significant increase in abnormal forms and testicular index. Sleep deprivation was linked to alteration of the quality of sperm [9, 14], however the precise mechanism has not been elucidated. It could be possible that the associated inflammatory and oxidative stress mediators play an injurious role with consequent reduction in sperm quality [26, 27]. Vitamin C has been shown to attenuate male reproductive dysfunction in diabetic rats [28].Vitamin C has also been reported to improve sperm count, motility, progression, viability and anomalies in rats subjected to forced swimming stress [22]. These effects were mainly attributed to the antioxidant and the testosterone increase properties of vitamin C. In our hands vitamin C countered SD-induced injurious effects on sperm characteristics, testis weight and testicular index. We then went to validate the underlying mechanisms, studying the possibility of anti-oxidant, anti-inflammatory, hormone and gene modifying effects.

SD induces intense alterations in the regulatory endocrinal axes, including the hypothalamic-pituitary-adrenal (HPA) axis. In the present study SD resulted in significant increase in serum cortisol and corticosterone levels, while it caused significant decrease in serum testosterone level. Vitamin C opposed the SD-induced hormonal alterations. Despite the stress modality, stress-induced increase in corticosterone and decrease in testosterone levels have been reported [9, 29]. In fact, the decrease in testosterone concentration was attributed to the increase in corticosterone level, as part of the stress-induced activation of the HPA axis, resulting in inhibition of the hypothalamic-pituitary-gonadal (HPG) axis [30]. Elevated corticosterone levels not only decreases testosterone production by Leydig cells, it also induces Leydig cells apoptosis [31, 32]. It has been reported that a negative relationship exists between cortisol and testosterone. Elevated cortisol levels were associated with decreased testosterone levels during exercise or even in disease status such as ischemic heart disease [33, 34]. Vitamin C supplementation has also been reported to attenuate cortisol responses following psychological or physical stressors [35]. Vitamin C is secreted from the adrenals in response to adrenocorticotrophic hormone (ACTH), representing a hormone-regulated paracrine secretion of vitamin C as part of the stress response [36]. Interestingly, and in support to our findings, supplementation with vitamin C attenuated the increase in the blood cortisol, adrenaline, interleukin-10 (IL-10) and interleukin-1 receptor antagonist (IL-1Ra) levels following ultra-marathon running [37]. Furthermore, vitamin C was shown to reduce corticosterone level in non-adrenalectomized rats alleviating stress-related behavior [38].Hence, we could imply that in our study vitamin C boosted testosterone concentration and thereby, improved SD-induced diminution in sperm quality.

It is well documented that oxidative stress is implicated in male factor infertility. In the present study, sleep deprived male rats showed higher serum and testicular tissue levels of MDA, while they had lower serum TAC and testicular tissue GSH and GPx levels when compared to the control group. Administration of vitamin C significantly attenuated sleep deprivation induced alteration in oxidative stress markers. A bidirectional relationship between sleep deprivation and oxidative stress has been documented [39–41]. Previous data showed evidence that the pathophysiology of male infertility was highly influenced by impairment in seminal antioxidant and lipid peroxidation status. Lifestyle stress, reduces male fertility; an increasing number of cases of male infertility are thought to be primarily due to oxidative stress [42]. MDA serves as an index of lipid peroxidation and a marker of oxidative stress, and could serve as a diagnostic tool for of infertility in asthenozoospermic patients [43, 44]. MDA level in seminal plasma has been reported to be negatively correlated with sperm viability, motility, morphology and concentration [44]. On the contrary, TAC levels were positively associated with sperm concentration, motility, and morphology [45]. GPx can be considered as a predictive measure for fertilization capacity. In fact, GPx is thought to be essential for structural integrity of spermatozoa, and a significant determinant of sperm motility and viability. Alteration in the content of GPx, irrespective of the cause, is negatively correlated with fertility-related parameter [46]. Glutathione (GSH) synthesis is induced in cells exposed to oxidative stress as an adaptive process. The relationship of GSH enzymatic system with oxidative stress in the ejaculate has gained much attention and the regulation of its activity in the semen has been suggested as a therapeutic strategy. Interestingly, intracellular sperm GSH system is altered in infertile men, which seems to be linked to sperm morphology. The quest to find novel antioxidants and/or combinations developed for safe and efficient treatment of oxidative stress induced infertility is likely to continue. Almost three decades ago, the antioxidant efficacy of vitamin C was shown to be effective for the treatment of sperm oxidative stress in smokers [47]. Since then, only few studies were conducted to confirm this finding. Indeed, vitamin C seems indispensible protector of semen from ROS; semen samples with excess ROS where found to be correlated with very low vitamin C concentrations [48]. Our results support the idea that vitamin C could be an efficient therapeutic option for the treatment of oxidative stress caused by sleep deprivation, and apparently, other environmental stressors via its potent antioxidant properties.

Since sleep deprivation can cause a state of inflammation [49], it was relevant to study the possible effects of inflammation on the male reproductive system. Inflammation has been known to affect the twin testicular functions; steroidogenesis and spermatogenesis. Marked decreases in the circulating levels of luteinizing hormone and testosterone were detected during inflammatory states [50]. Indeed, testicular inflammatory disorders leading to impairment of spermatogenesis are considered to be a primary cause for male infertility. The testis is considered as an immune privileged organ, nevertheless, toxic agents and inflammation may overwhelm immune suppressor mechanisms resulting in autoimmune reactions against spermatic antigens. Consequently, this may result in aspermatogenesis and infertility [51]. In the present study, sleep deprivation resulted in significant rise in IL-6 and Il-17, which could be countered by the treatment with vitamin C. Nevertheless, some cytokines such as IL-1, and IL-6 can also produced by Leydig and Sertoli cells [25]. Consequently, it is possible that cytokines may act not only towards somatic cells, but also towards the germ cells both in an autocrine and paracrine fashion. It is possible that cytokines may act during spermatogenesis, sperm maturation, sperm transport, and even during the fertilization process itself. Cytokines such as interleukins and tumor necrosis factors are involved in signal transduction during inflammatory states [26]. Despite the existing controversy regarding the role of cytokines in fertility, our results were in agreement with previously published data reporting that significantly elevated IL-6 levels were found in infertile patients and revealed an apparent negative correlation with sperm number. Moreover, infertile patients with varicocele exhibited an elevated levels of IL-6 [52, 53]. Excess IL-17 is commonly associated with different types of inflammation, and as for our study, IL-17 serum levels were elevated in the sleep deprived male rats. It has been reported previously that LI-17 and its signaling pathway were highly expressed in mice testis exposed to high fluoride [51]. IL-17 was found to be critically involved in male patients with azoospermic testis with chronic inflammation. To our knowledge, this could be the first report on the effect of Vitamin C on serum IL-6 or IL-17 vis-à-vis male fertility.

In the present study, vitamin C had significantly countered the sleep deprivation-induced increased expression of testicular NF-κβ and decreased expression of testicular Nrf2 genes. NF-κβ can be activated by a multiplicity of stimulants including ROS through the phosphorylation of the inhibitory kappa B (IκB) by IκB kinases. NF-κβ is known to activate several genes including the inducible nitric oxide synthase (iNOS), resulting eventually in excessive generation of nitric oxide (NO) [54]. NO, if oxidized, generates reactive NO species, which might behave similarly to ROS. It has been reported previously that NO could enhance cellular injury by decreasing intracellular GSH levels [55]. Nrf2, a redox sensitive transcription factor, is a fundamental contributor to oxidative stress homeostasis [56]. Nrf2 is involved in the regulation of the synthesis and conjugation of glutathione (glutamate-cysteine ligase catalytic subunit), and antioxidant proteins responsible for the detoxification of ROS [57]. It was reported previously that Nrf2 expression is significantly lower in semen of men with low sperm motility [58]. Nrf2 plays an important role in preventing oxidative disruption of spermatogenesis. In fact, Nakamura et al. demonstrated that male Nrf2 knockout mice (Nrf2−/−) have decreased fertility compared to the wild type. They also reported that Nrf2−/− male mice had elevated levels of testicular and epididymal lipid peroxidation, prominent testicular germ cell apoptosis, and reduced antioxidants levels compared to wild type male mice [59].

Histopathological and immunostaining studies demonstrated that vitamin C has protective effects at the structural level. Sleep deprivation resulted in disruption of the normal morphology of spermatogonia and occasionally apoptosis. Treatment with vitamin C retained much of the normal morphology and regularity of the seminiferous tubules and the different stages of spermatogenesis. Preservation of spermatogenesis was further supported by enhancement of the PCNA immunostaining in the sleep deprived vitamin C treated rats. PCNA could serve as a biomarker for spermatogenesis [60].

## Conclusion

Sleep deprivation, whatever the cause is, has serious effects on male fertility. We showed here that vitamin C maintained testicular structure and enhanced testicular function in sleep deprived rats. Vitamin C counteracted sleep deprivation dependent alteration in sperm analysis, hormonal levels, and inflammatory and oxidative stress biomarkers. Vitamin C modified the sleep deprivation-dependent altered Nrf2 and NF-κβ gene expression. Consequently, vitamin C could be a potential fertility enhancer in opposition to lifestyle stresses.

## Data Availability

Data supporting findings are presented within the manuscript.

## Abbreviations

ACTH: Adrenocorticotrophic hormone
GAPDH: Glyceraldehyde-3-phosphate dehydrogenase
GnRH: Gonadotropin releasing hormone
GSH: Glutathione
GSH-Px: Glutathione peroxidase
HPA: Hypothalamic-pituitary-adrenal axis
IL-17: Interleukin 17
IL-6: Interleukin 6
MDA: Malondialdehyde
NF-κβ: Nuclear factor kappa beta
Nrf2: Nuclear factor (erythroid-derived 2)-like 2
PCNA: Proliferating cell nuclear antigen
PUFA: Polyunsaturated fatty acids
ROS: Reactive oxygen species
SD: Sleep deprivation
TAC: Total antioxidant capacity
